# Construction of an infectious clone of Zika virus stably expressing an EGFP marker in a eukaryotic expression system

**DOI:** 10.1186/s12985-021-01622-z

**Published:** 2021-07-19

**Authors:** Jing Gao, Jiayi Chen, Weizhi Lu, Jintai Cai, Linjuan Shi, Wei Zhao, Bao Zhang

**Affiliations:** 1 Biosafety Level 3 Laboratory, School of Public Health, Southern Medical University, Guangzhou, 510515 China; 2 Guangdong Provincial Key Laboratory of Tropical Disease Research, School of Public Health, Southern Medical University, Guangzhou, 510515 China

**Keywords:** Zika virus, EGFP marker, Recombinant virus, Eukaryotic expression

## Abstract

**Background:**

Zika virus is becoming one of the most widely transmitted arboviruses in the world. Development of antiviral inhibitor and vaccine requires an experimental system that allows rapid monitoring of the virus infection. This is achievable with a reverse genetic system. In this study, we constructed an infectious clone for Zika virus that stably expressing EGFP.

**Methods:**

A PCR-mediated recombination approach was used to assemble the full-length Zika virus genome containing the CMV promoter, intron, EGFP, hepatitis delta virus ribozyme, and SV40 terminator sequence for cloning into the pBAC11 vector to produce recombinant pBAC-ZIKA-EGFP. ZIKA-EGFP virus was rescued by transfection of pBAC-ZIKA-EGFP into 293T cells. The characterization of ZIKA-EGFP virus was determined by qPCR, plaque assay, CCK-8, and Western blot.

**Results:**

Rescued ZIKA-EGFP virus exhibited stable replication for at least five generations in tissue culture. ZIKA-EGFP can effectively infect C6/36, SH-SY5Y and Vero cells, and cause cytopathic effects on SH-SY5Y and Vero cells. The inhibition of ZIKA-EGFP by NF-κB inhibitor, caffeic acid phenethyl ester was observed by fluorescence microscopy.

**Conclusion:**

Our results suggested that Zika virus infectious clone with an EGFP marker retained it infectivity as wide-type Zika virus which could be used for drugs screening.

**Supplementary Information:**

The online version contains supplementary material available at 10.1186/s12985-021-01622-z.

## Background

Zika virus was transmitted mainly by the mosquito and has become epidemic worldwide. Zika virus was first discovered in the Zika Forest in Uganda in 1947, and an outbreak of Zika virus in the Americas in 2015 caused a global public health emergency [1–4]. Approximately 80% of Zika infections are asymptomatic, with the most common symptoms including fever, arthralgia, rash, myalgia, edema, vomiting, and non-purulent conjunctivitis [5, 6]. The virus can infect the placenta and blood–brain barrier endothelial cells, neurons, and neural stem cells [7, 8] and is closely associated with diseases, such as encephalitis, fetal microcephaly, and Guillain–Barré syndrome [9–11]. Similar to other flaviviruses, the Zika genome encodes an open reading frame containing three structural proteins (C: capsid; prM: precursor of membrane; and E: envelope) that comprise the viral particles and seven genes encoding non-structural (NS) proteins (NS1, NS2A, NS2B, NS3, NS4A, NS4B, and NS5) that participate in RNA replication [12].

Recent studies show that some mutations of the Zika virus are related to host adaptation [13–16], however, the pathogenic mechanism of the virus has not been fully elucidated, and there is no available vaccine or therapeutic. The reverse genetic of Zika virus with luciferase or fluorescent protein have been described [1, 17, 18]. Mutso et al*.* reported that recombinant Zika virus carried maker genes such as nanoluciferase, enhanced green fluorescent or mCherry, which inserted between two copies of the capsid protein gene were genetically stable for at least four in vitro passages [18]. This system allows rapid monitoring of the infected cells, which provides a great convenience for the screening antiviral inhibitors.

This study describes construction of full-length infectious clone of Zika virus in a eukaryotic expression system that can be used to produce the virus along with stable expression of the enhanced green fluorescence protein (EGFP) marker. In some previous methods, recombinant Zika virus carrying EGFP marker cannot be stably passaged [1, 18], so it is often required to perform multiple virus rescue experiment. Virus rescue is often time-consuming and expensive, which required multiple attempts to obtain sufficient amount of rescued virus. In this study, we have established a Zika virus infectious clone with EGFP reporter which exhibited similar replication competency as wild-type ZKC2/2016 strain. Due to its inherent stability, we are able to produce high virus titer with only a single virus rescue experiment.

## Materials and methods

### Cells, virus and reagents

The Vero cells, 293T cells, and C6/36 cells originally held in our laboratory were cultured at 37 °C with 5% CO_2_ in Dulbecco’s modified Eagle’s medium (DMEM) supplemented with 10% fetal bovine serum (FBS; Gibco, Gaithersburg, MD, USA). SH-SY5Y cells (a thrice cloned subline of the neuroblastoma cell line SK-N-SH) obtained from KeyGEN BioTECH (NanJing, JiangSu, China) and were cultured at 37 °C with 5% CO_2_ in DMEM/F12 supplemented with 10% FBS. ZIKA-WT (ZKC2/2016, GenBank Accession NO.: KX253996), provided by the Institute of Virology of Guangdong Provincial Center for Disease Control and Prevention, was amplified in Vero cells.

Anti-Zika E and NS1 antibody were obtained from GeneTex (San Antonio, TX, USA); anti-EGFP antibodies (Abcam, Cambridge, MA, USA); DAPI, Phycoerythrin (PE) labelled goat-anti-mouse antibody, anti-β-actin, anti-mouse and anti-rabbit secondary antibodies and enhanced chemiluminescence (ECL) Horseradish Peroxidase (HRP) substrate (Bioworld, Minneapolis, MN, USA); pEGFP-N1 vector, Infusion kit, TRIzol, PrimeScript RT reagent kit, Besstar™ qPCR Master Mix, TB Green™ Premix (Takara, Dalian, China); pBAC11 (Addgene, Watertown, MA, USA); restriction enzymes *Sfo*I and *Pac*I, DH10B cells (New England Biolabs, Ipswich, MA, USA); Lipofectamine 3000 (Thermo Fisher Scientific, Waltham, MA, USA); 0.45-μm membrane filter, (Pall Corporation; Port Washington, NY, USA); polyvinylidene difluoride (PVDF) membrane (Millipore, MA,USA); bovine serum albumin (BSA), IPA lysis buffer (Genshare, Xian, China).

### Construction of a full-length infectious clone of Zika virus

**Fig. 1:**
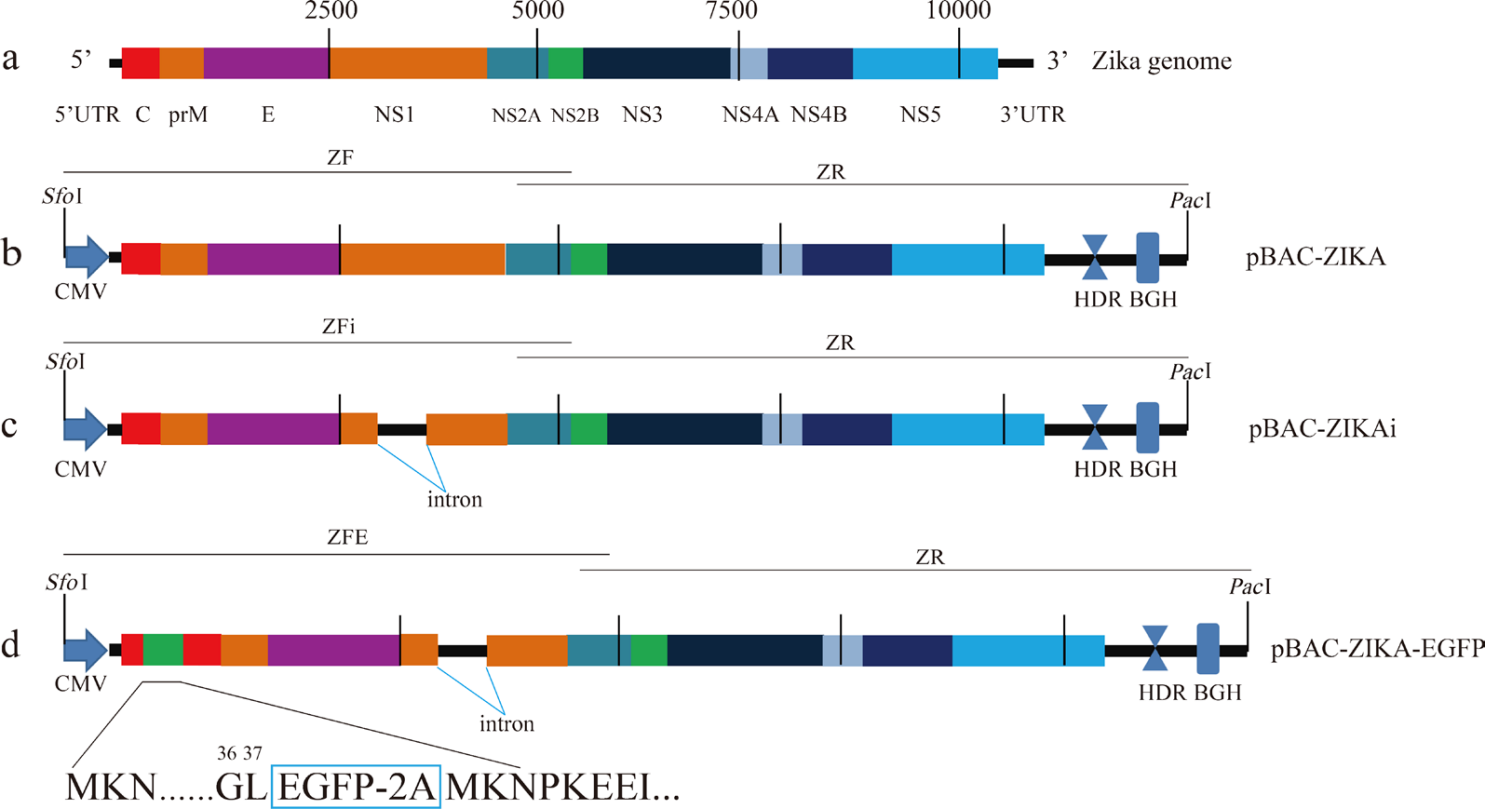
**a** Genomic structure of the Zika virus. **b** Schematic diagram showing the structure of pBAC-ZIKA. CMV, HDV, and BGH denote the CMV promoter sequences, the hepatitis delta virus ribozyme sequence, and the bovine growth hormone terminator sequence, respectively. *Sfo*I and *Pac*I are restriction endonucleases. pBAC-ZIKA was constructed by recombination of the ZF and ZR fragments using the restriction-digested vector. **c** Schematic diagram showing the structure of pBAC-ZIKAi. The ZFi fragment was generated by inserting the intron sequence into the genomic sequence of the Zika virus downstream of 3128 nt, followed by its recombination with ZR with the restriction-digested vector to generate pBAC-ZIKAi. **d** pBAC–ZIKA–EGFP was constructed by inserting the EGFP-2A gene downstream of the 37th codon of the Zika virus sequence

As shown in Fig. 1, the cytomegalovirus (CMV) promoter sequence was amplified from pEGFP-N1 vector with primers (ZCMVF: 5′-*AAGGAGAAAATACCGCATCAGGC*GCGTTGACATTGATTATTGACTAG-3′ and ZCMVR: 5′-CTGTCGCAGTCTGATTCACACAGATCAACAACTTGTTAGCCAGAGAGCTCTGC-3′, the *Italics* sequence is same with vector of pBAC11, the Underline sequence is Zika viral genomic sequence 1-31nt) synthesized by Guangzhou IGE Biotechnology Co., Ltd. (Guangzhou, China), then fused to the fragment of the Zika viral genomic sequence 1–5908nt according to the strain of ZKC2/2016 with primers (Z1F: 5′-GCAGAGCTCTCTGGCTAACAAGTTGTTGATCTGTGTGAATCAGACTGCGACAG-3′ and Z1R: 5′-GGCTTTAGGCATCTCCTGGAATCTATG-3′) using overlap polymerase chain reaction (PCR) method in order to generate the 5' half sequence of the Zika virus infectious clone (ZF). The fusion fragment of hepatitis delta virus (HDV) ribozyme sequence (688-771nt according to GenBank Accession NO.MK890235.1) with 5′ additional 10707-10807nt of Zika genomic sequence and bovine growth hormone terminator sequence (1027-1279nt according to pCDNA3.1- vector) with 3′ additional 150 bp before *Pac*I site of pBAC11 vector were synthesized by Guangzhou IGE Biotechnology Co., Ltd., then ligated to the Zika viral sequence (5735–10807nt) using overlap PCR method with primers (Z2F: 5′-TTGTCTGACAAAGGCTGGAAAACGGGTCATACAG 3’ and Z2R: 5’ CCCCGCGGACCCCGGTTTAATTAACAGCTGGTTCTTTCCGCCTCAGAAG-3′; the Underline sequence is same with pBAC11 vector) to generate the 3' half sequence of the clone (ZR). Recombination reaction of three fragments of ZF, ZR and pBAC11 vector previously digested with restriction enzymes (*Sfo*I and *Pac*I) was performed using Infusion kit (Takara, Dalian). The recombinant of pBAC-ZIKA were transformed into DH10B cells, screened with 20 μg/ml chloramphenicol LB agar plate and sequenced by Guangzhou IGE Biotechnology Co., Ltd. (Fig. 1b).

The intron sequence (derived from the pCI-neo vector plasmid according to GenBank Accession NO.KU870446.1) synthesized by Guangzhou IGE Biotechnology Co., Ltd. was inserted into the ZF sequence of the Zika virus (between 3128 and 3129nt) using overlap PCR method as pBAC-ZIKA mentioned to generate the sequence of the ZFi fragment (the G-to-A mutation introduced at nt3128 was used as a genetic modified marker). The ZFi and ZR fragments were recombined with pBAC11 vector digested to generate recombinant pBAC-ZIKAi (Fig. 1c).

The EGFP-2A gene (fusion sequence of EGFP gene from pEGFPN1 vector and 2A peptide of foot and mouth disease virus) was inserted downstream of the 37th codon of the Zika virus using the above-described methods to produce the fragment of ZFE with 2A sequence adjoined to first codon of Zika full-length coding-region sequence. Briefly, ZFE fragment was achieved by splicing overlap extension PCR method with three PCR fragments (first was amplified from template of pBAC-ZIKAi with primers of ZCMVF and E1 5′-GCTTGAAGAGGCTGCCAGCCGGACTTCTGCTG-3′, second was amplified from template of pEGFPN1 with primers of E2F 5′-AGTCCGGCTGGCAGCCTCTTCAAGCATGGTGACAAGGGCGAGGAGC-3′ and E2R 5′-CGTCACCGCATGTTAGAAGACTTCCTCTGCCCTCCTTGTACAGCTCGTCATGCCGA-3′, third was amplified from template of pBAC-ZIKAi with primers of E3F 5′-GAAGTCTTCTAACATGCGGTGACGTGGAGGAGAATCCCGGCCCTATGAAAAACCCAAAAAAGAAATC-3′and Z1R. Fragments of ZFE, ZR and pBAC11 digested with *Sfo*I and *Pac*I were recombined with Infusion kit to construct pBAC-ZIKA-EGFP (Fig. 1d).

The sequences of 2A peptide, intron, HDV ribozyme and bGH polyA signal were listed in the Additional file 1: S1.

### Rescue of recombinant Zika virus

293T cells were seeded in 6-well plates and transfected with 5 μg/well of the pBAC–ZIKA–EGFP or pBAC–ZIKAi plasmid using Lipofectamine 3000 according to the manufacturer’s instruction. The supernatant containing P0 generation of the ZIKA-EGFP or ZIKAi virus were harvested 7 days post transfection, passed through a 0.45-μm membrane filter, and stored at − 20 °C. Vero cells were infected with P0 recombinant virus at a multiplicity of infection (MOI) of 0.1. At 4 to 6 days post-infection, supernatant containing the P1 generation was collected, and Vero cells were re-infected with the P1 virus at a MOI of 1.0 in order to produce viral generations P2, P3, P4, P5.

Total RNA from Vero cells infected with ZIKA–EGFP (generation P5) was extracted using TRIzol, and *EGFP* expression was examined by PCR using primer 1 (5′-AGTTGTTGATCTGTGTGAATCAGACTG-3′) targeting the Zika sequence and primer 2 (5′-CTTGTACAGCTCGTCCATGCCGAGAGTG-3′) targeting the *EGFP* sequence. Cytopathic changes on Vero cells and expression of green fluorescence were monitored.

### Immune fluorescence assay

5 × 10^5^ of Vero cells in a 6-well plate were infected with virus of ZIKA-WT and ZIKA-EGFP (generation P5) at a MOI = 0.1. At 48 h post-infection, cells were fixed with 95% ethanol for 20 min, immune-stained with anti-E protein antibody (4G2, 1:200) for overnight at 4 °C. After 5 times washing, the cells were stained with PE-labelled goat-anti-mouse antibody (1:300), then stained with DAPI. Cells were observed by fluorescent microscopy (200×).

### Western blot analysis

Western blot was used to measure levels of EGFP, NS1 and E proteins in Vero cells infected with ZIKA-WT, ZIKAi or ZIKA–EGFP (generation P5). Briefly, infected Vero cells in 6-well plates were harvested by addition of RIPA lysis buffer. Forty micrograms of proteins were electrophoresed in SDS–polyacrylamide gel electrophoresis gel and transferred onto a PVDF membrane. PVDF membranes were blocked with 3% BSA buffer in tris buffered saline/0.1% Tween 20 for 2 h at room temperature and then incubated with the following primary antibodies: anti-β-actin (1:5000); anti-Zika E protein (1:2000); anti-Zika NS1 (1:2000); anti-EGFP (1:5000) overnight at 4 °C with gentle agitation. Then the membranes were incubated with HRP-conjugated secondary antibodies for 1 h at room temperature with ECL reagent for exposure. Protein band were detected using a Luminescent image Analyzer (Tanon-5200, Shanghai, China).

### Fluorescence-based quantitative PCR (qPCR)

Total RNA was extracted using TRIzol in accordance with the manufacturer’s instructions. Thereafter, 1 μg of total RNA was reverse-transcribed to cDNA using PrimeScript RT reagent kit. qPCR was performed using Bestar™ qPCR Master Mix with Zika E gene sense primer: 5′-CVGACATGGCTTCGGACAGY-3′, antisense primer: 5′-CCCARCCTCTGTCCACYAAYG-3′, and probe: 5′-AGGTGAAGCCTACCTTGACAAGCARTCA-3′ according to the following steps: preincubation (95 °C for 120 s); 2-step amplification (10 s at 95 °C, 30 s at 60 °C for 40 cycles) with data collection at the end of 60 °C; cooling (37 °C for 30 s). qPCR was performed using TB Green™ Premix with GPADH sense primer: 5′-GTCAAGGCTGAGAACGGGAA-3′, antisense primer 5′-AAATGAGCCCCAGCCTTCTC-3′ according to the following steps: preincubation (95 °C for 30 s); 3-step amplification (15 s at 95 °C, 15 s at 55 °C, 15 s at 72 °C for 40 cycles) with data collection at the end of 72 °C; melting (95 °C for 10 s, 65 °C for 30 s, 97 °C for 1 s); cooling (37 °C for 30 s). Zika viral mRNA expression levels were normalized to those of GAPDH using the 2^−ΔΔCt^ method. The results are expressed as expression fold changes. qPCR can detect more than 1copy/μL DNA determined by standard curve with plasmid.

### Viral replication ability

Growth kinetics of ZIKA–EGFP was compared with ZIKA-WT in vitro. Briefly, Vero cells were seed in 12-well plates (2 × 10^5^ cells/well). 12 h later, both viruses were inoculated into individual wells (MOI = 0.1). Supernatants and cells were collected at 12 h, 24 h, and 48 h post-infection, and total RNA was extracted and reverse transcribed. The replication of viral RNA in cells was measured using qPCR [19]. An assay to determine the 50% tissue culture infectious dose (TCID_50_) with Vero cells was used to determine viral titer in the supernatant, with TCID_50_ values calculated according to the Reed–Muench method.

### Viral toxic effects on cells

ZIKA-WT and ZIKA-EGFP were used to infect SH-SY5Y cells at a MOI of 1.0. At 48 h and 72 h post-infection, cell viabilities were measured using a Cell Counting Kit-8 (CCK-8; Dojindo, Shanghai, China) assay. Cytopathic effects were observed using a microscope (Eclipse E200, Nikon, Tokyo, Japan).

Plaque assay was used to determine the cytopathic effect of ZIKA-WT and ZIKA–EGFP. Vero cells were seeded in 24-well plates at 5 × 10^4^ cells/well, and the following day, an appropriate viral titer was used to infect the cells. At 2-h post-infection, 2% carboxymethylcellulose was used to overlay the cells, and after 6 days, Crystal Violet staining was performed to observe plaque sizes.

### Infectivity to the C6/36 cells

5 × 10^4^ C6/36 cells/well were seeded in 24-well plates. After 24 h, cells were infected by ZIKA-EGFP with MOI = 0.1. At 48 h, 72 h and 144 h post-infection, cells were observed by fluorescent microscope (Eclipse E200, Nikon, Japan).

### Inhibitory effect of caffeic acid phenethyl ester (CAPE; Selleck, Shanghai, China) on ZIKA–EGFP Viral Replication

Vero cells were infected with ZIKA–EGFP and ZIKA–WT (MOI = 0.1) and cultured with 2 ml media containing 10 μM CAPE for 48 h. Relative expression levels of the viruses were measured using qPCR. Additionally, fluorescence intensity was observed under a fluorescence microscope.

### Statistics analysis

All experiments were repeated at least three times. Data were presented as mean ± SEM. Results were analyzed using Graphpad Prism (GraphPad Software Inc., La Jolla, CA). Comparison between experimental groups was made by Student’s t-test and one-way ANOVA. A value of *P* < 0.05 was considered statistically significant. (*P < 0.05, **P < 0.01, ***P < 0.001, ^n.s^*P* > 0.05).

## Results

### Production of rescued virus

**Fig. 2:**
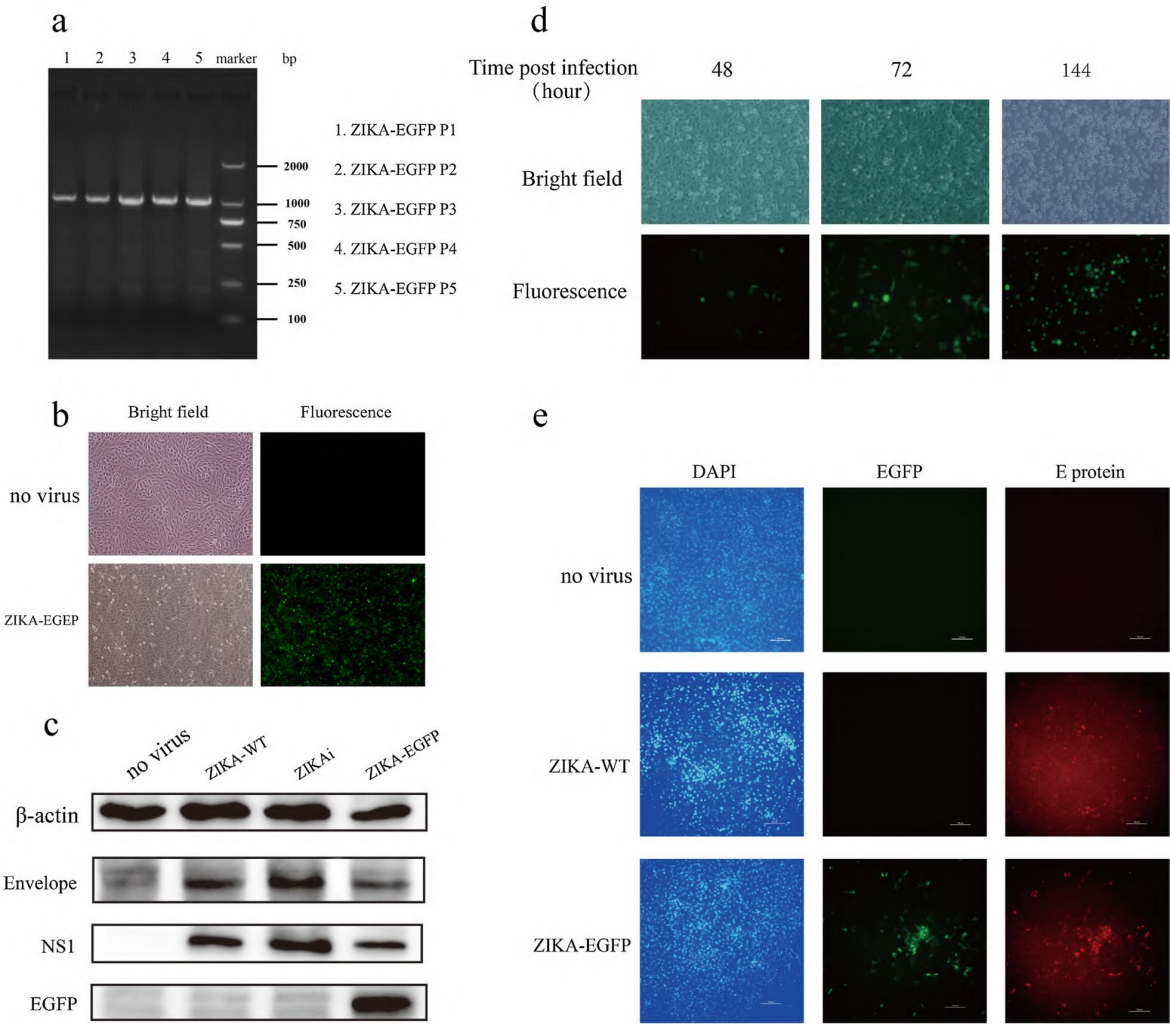
Rescue of recombinant ZIKA–EGFP. **a** Detection of *EGFP* by electrophoresis. (1–5 lanes are generation p1-p5 of recombinant ZIKA-EGFP, respectively) **b** Fluorescence of Vero cells infected with the ZIKA–EGFP (generation P5) for 48 h (×200). **c** Western blot detection of E gene, NS1 gene, and EGFP gene levels in Vero cells infected with the ZIKA-WT, ZIKAi (generation P5), and ZIKA–EGFP (generation P5) for 48 h. Western blot analysis was repeated three times and representative result was exhibited. **d** Fluorescence of C6/36 cells infected with ZIKA–EGFP at different time points. **e.** The localization of EGFP and E protein for ZIKA-EGFP in Vero cells (×200)

The pBAC–ZIKA–EGFP clone generated as described in Fig. 1 was used to transfect 293T cells, supernatant was collected at 7-days post-transfection and used to infect Vero cells. The viruses were passaged every 4–6 days. Total RNA was extracted from the cells after infection and identified *EGFP* expression by PCR, demonstrating the stability of the ZIKA–EGFP viral sequence following passaging (Fig. 2a). At 48-h post-infection with ZIKA–EGFP (generation P5), green fluorescence was clearly visible under fluorescence microscope (Fig. 2b), at the same time, some cells could be observed rounded, crinkled, and even shed in the bright field of microscopy, which suggested that recombinant ZIKA-EGFP could induce cytopathic effect in Vero cells (Fig. 2b). We used Western blot experiment to confirm that ZIKA-EGFP (generation P5) stably express EGFP using NS1 and E proteins of Zika virus as references in Vero cells at 48 h post-infection. The rescued ZIKAi virus (generation P5) expressed NS1 and E proteins, which indicated that rescued ZIKAi virus by eukaryotic expression system was steady (Fig. 2c). Zika virus is transmitted by Aedes mosquitoes. Therefore, we want to know if ZIKA-EGFP can successfully infect C6/36 cells. We can observe more cells express EGFP over time when C6/36 cells were infected with ZIKA–EGFP, suggesting that ZIKA-EGFP can also infect mosquito cell line and replicate in cells (Fig. 2d). Moreover, ZIKA-EGFP could be passaged at least six generations with EGFP expression (data not shown). To further confirmed that the genome ZIKA-EGFP stability, EGFP and E protein expression were observed by immune fluorescence (Fig. 2e) which showed that EGFP and E protein had colocalized in the ZIKA-EGFP group.

### Viral replication ability

**Fig. 3:**
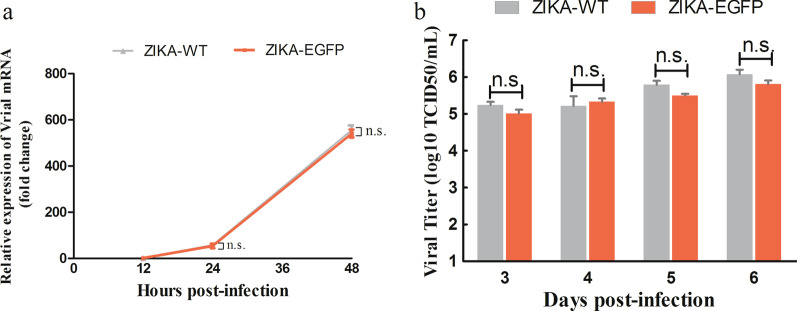
Viral kinetics of ZIKA–WT and ZIKA–EGFP in Vero cells. Vero cells were infected with ZIKA–WT and ZIKA–EGFP, and **a** intracellular viral RNA expression was detected at various time points by qPCR. **b** Viral titer in culture supernatant was determined by TCID_50_ assay. All experiments were repeated three times and each group contained three replicates

Levels of viral RNA in ZIKV-EGFP infected Vero cells (MOI = 0.1) at 12 h, 24 h, and 48 h post-infection were measured using qPCR. The results showed that ZIKA–EGFP viral RNA could be amplified in Vero cells and was comparable with ZIKA-WT (Fig. 3a), which revealed that no significant difference in the replication kinetic between ZIKA-EGFP and ZIKA-WT. Culture supernatants were then collected and subjected to TCID_50_ assay. Our data showed similar titers between ZIKA–EGFP and ZIKA–WT (Fig. 3b), indicating similar viral replication kinetic between ZIKA–EGFP and ZIKA–WT.

### Viral cytopathic effects on cells

**Fig. 4:**
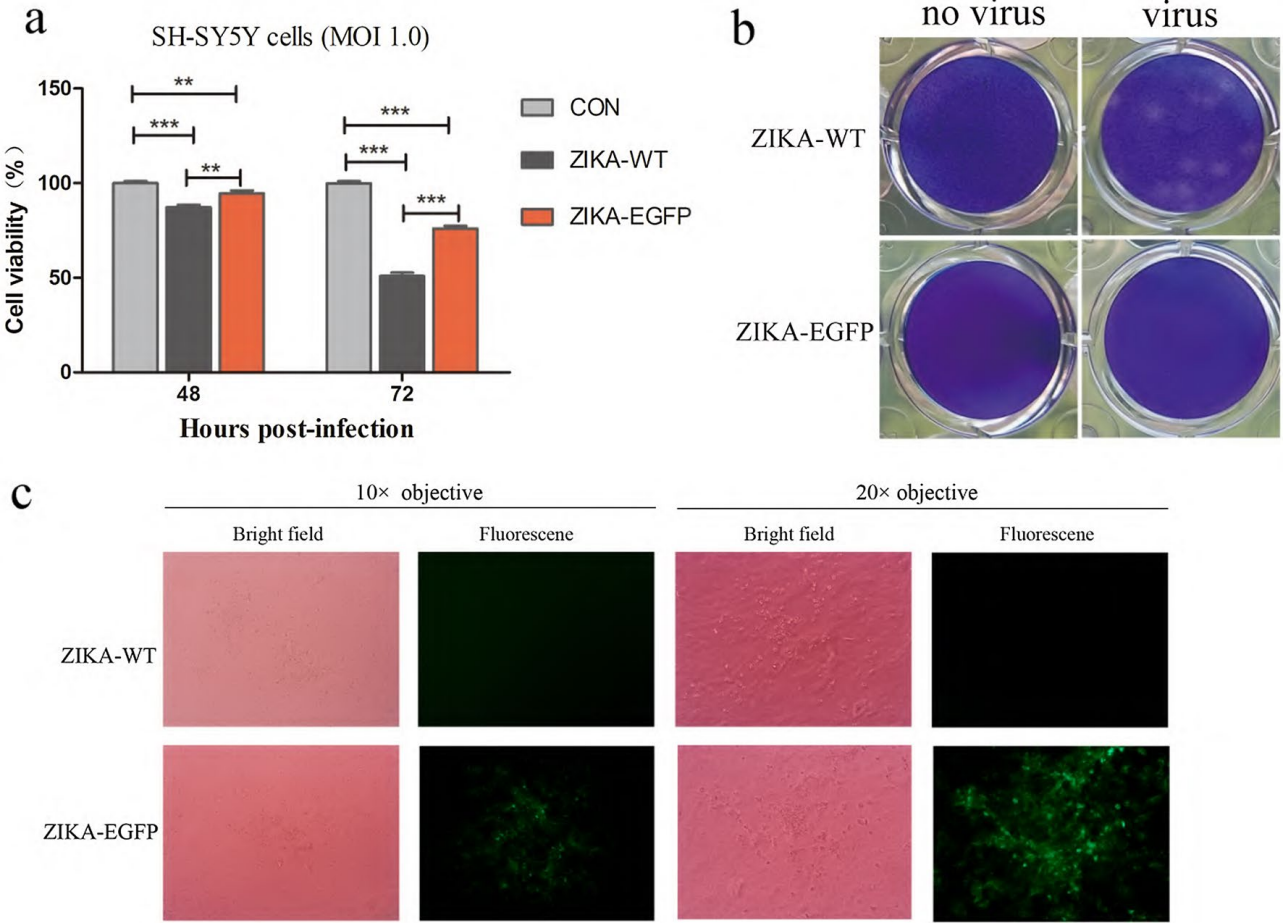
Comparison of ZIKA–EGFP and ZIKA–WT toxic effects cells. **a** SH-SY5Y cells were incubated with ZIKA–WT and ZIKA–EGFP (MOI = 1.0), and cell viability was determined by CCK-8 assay at 48- and 72-h post-infection. **b** Cytopathic effect was compared by plaque assays with Vero cells. **c** Fluorescent cells aggregated due to the cytopathic effects associated with ZIKA–EGFP in Vero cells. CCK-8 tests were repeated three times and each group contained six replicates. **P < 0.01, ***P < 0.001

Neurotropism of Zika virus is associated with encephalitis, fetal microcephaly, and Guillain–Barré syndrome closely. We include SH-SY5Y cell line, a subline of neuroblastoma, to study the replication competency of ZIKA-EGFP. After infection of SH-SY5Y cells with ZIKA–EGFP and ZIKA–WT (MOI = 1.0) for 48 h, we observed prominent cytopathic effects (data not shown). Additionally, CCK-8 assays showed that cells viability of ZIKA-EGFP-infected cells decreased significantly in time-dependent manner, but ZIKA-EGFP cytopathic effect was weaker than ZIKA–WT, with cells viability of 75% and 49% for ZIKA-EGFP and ZIKA-WT at 72 h post-infection, respectively (Fig. 4a). Plaque assay results showed that ZIKA–EGFP did not result in plaque formation in Vero cells, while ZIKA-WT led to the formation of prominent, perfectly round, and uniform plaques after 6 days post-infection (Fig. 4b). Green fluorescence cell clusters were observed indicating that ZIKA-EGFP did infect Vero cells (Fig. 4c).

### Inhibitory effect of CAPE on viral replication

**Fig. 5:**
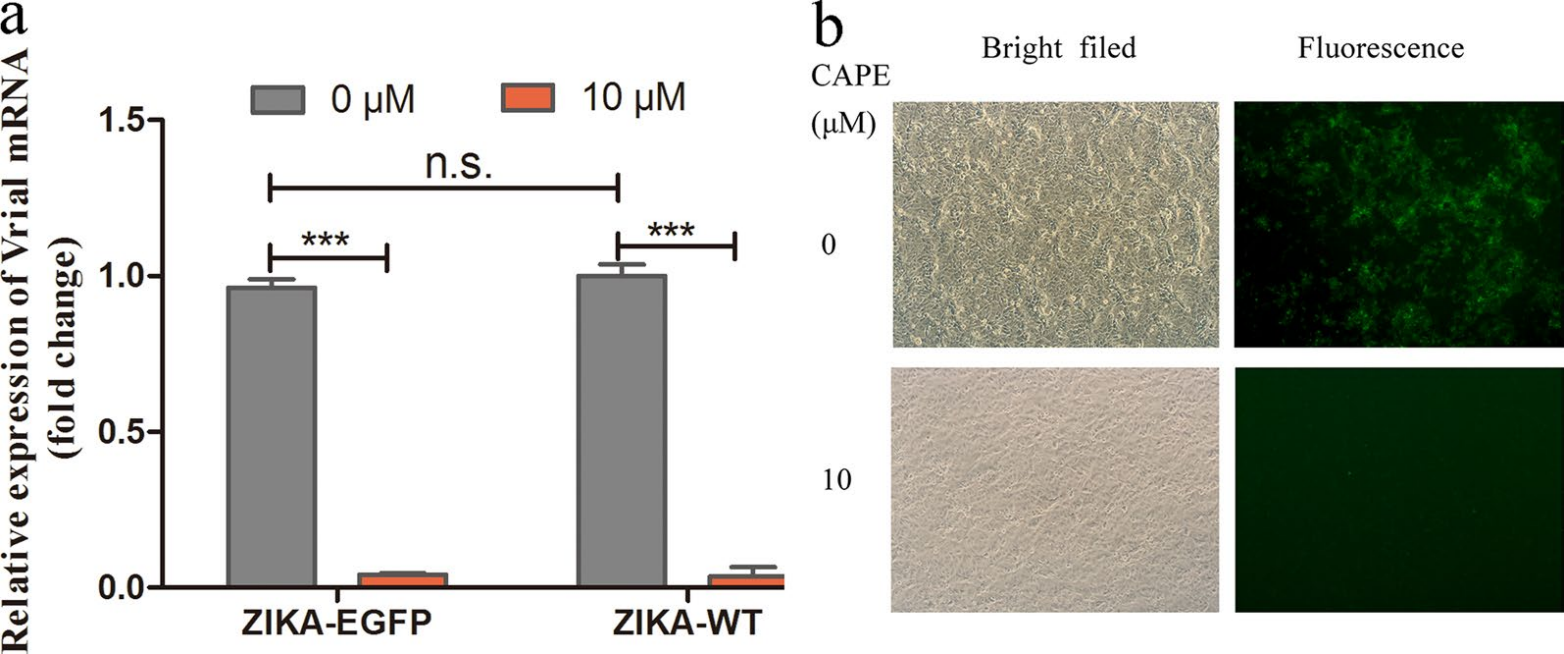
CAPE inhibition of ZIKA–EGFP infection. **a** Vero cells infected with ZIKA–WT and ZIKA–EGFP at a MOI of 0.1 were cultured in the presence and absence of 10 µM CAPE for 48 h, followed by detection of Zika viral E gene expression by qPCR. **b** Fluorescent cells were observed under a fluorescence microscope (×200). The qPCR assays were repeated three times and each group contained three replicates. ***P < 0.001, n.s., not significant

Vero cells infected with ZIKA–EGFP and ZIKA–WT (MOI = 0.1) were treated with 10 μM CAPE for 48 h. The qPCR results revealing that CAPE significantly inhibited viral replication (Fig. 5a). These findings were further confirmed by immunofluorescence assay (Fig. 5b).

## Discussion

The reverse genetics approach is an important method to study viral gene function and virulence, and as an important tool to screen for anti-Zika virus therapeutic. The presence of cryptic bacterial promoters in flaviviruses has increased the challenges associated with construction of infectious clones [20, 21]. The common solution to this issue is mutation of the cryptic promoter [22] or utilization of a single-copy plasmid-based bacterial artificial chromosome [20]. In this study, we used a bacterial artificial chromosome to reduce the toxicity of the viral DNA in order to obtain a full-length clone of Zika virus.

Infectious clones can be constructed in three types of systems. The first is the in vitro transcription system [23–25], where a full-length DNA clone is used as a template to transcribe viral mRNA molecules using T7 or SP6 RNA polymerase. The purified viral RNA is then used to transfect cells via electrotransfection or other methods to produce rescued viruses. This method requires highly pure mRNA and often expensive and time-consuming. The second approach involves use of eukaryotic systems [1, 25, 26], where a clone containing the full-length viral sequence is constructed and placed under the control of a eukaryotic promoter, such as CMV or SV40. Upon transfection of target cells with the plasmid, viral mRNA can be generated using the transcription machinery of the eukaryotic cells, enabling subsequent rescue of the viruses produced in the cells. This method generally does not require expensive reagents or instruments, is simple to perform, and the conditions are easily optimizable. However, the limitation of this method involves the need for transcription inside the nucleus during generation of viral mRNA. We failed to produce a rescued Zika virus because there was a fragment deletion of mRNA, which is probably related to abnormal RNA splicing. We then inserted an intron sequence to guide correct RNA splicing at 3128nt of Zika virus genome sequence which successfully to rescue virus. Avila-Perez et al. and Chen et al. used this method to construct infectious clones and did not observe incorrect pre-mRNA splicing, which could be due to sequence differences in different viral strain [20, 22]. The third approach is using recombination systems with overlapping fragments [27, 28]. In this system, the full-length DNA clone and the transcription-control elements are amplified as three fragments, which are then transfected into target cells via electrotransfection. The complete viral genome sequence is then generated in the cells by homologous recombination to subsequently produce the rescued viruses. This method can avoid the toxic effect of promoter of the cryptic bacterial, however, the transfection conditions need to be optimized, and the efficiency of homologous recombination can greatly affect viral production.

To rapidly screen drugs for inhibiting the viral replication and study the mechanism of replication and translation, Xie et al. constructed replication system that the chimeric replicon of Rluc gene and viral genome could stably passage, but not produce virus particles [29].

To facilitate investigations of Zika virus pathogenesis, previous studies attempted to include a marker, such as EGFP or luciferase at C25 [1], C33 [17], or other positions, in the Zika viral genome. However, most attempts did not lead to stable expression of the markers. Gadea et al.found that the recombinant ZIKV-GFP with EGFP gene at the downstream of the first 33 amino acids of Capsid protein (C33) was unstable and the virus would lose EGFP expression after 3 generation passages. Zou et al. found recombinant Luc-DENV-2 with RLuc gene inserted at first 38 amino acids of Capsid protein (C38) was stable for at least 5 generations [30]. Upon analysis of the secondary structure of the viral RNA genome of Zika virus and Dengue-2 virus, we found that Zika virus has similar structure with Dengue-2 on the 5′ and 3′ terminal of genomic sequence. These evidences indicated that Zika virus genome might exist sites for exogenous gene insertion which could stably passage. Dengue virus C38 (Leucine amino acid) locates in the middle of the sixth stem-loop structure. We designed the marker-insertion site at the C37 position (Leucine amino acid), which corresponds to the site of C38 of Dengue-2 virus. We proved that expression of EGFP is stable during passages in multiple dimensions including genome level, protein level and direct observation of green fluorescence. Our results showed that insertion of EGFP at the C37 position led to a ZIKA–EGFP viral sequence that remained stable in the genome for at least five generations or more, with no adverse effect on the range of cells that could be infected (the ZIKA–EGFP was able to infect Vero, SH-SY5Y, and C6/36). Additionally, we found that the viral-replication kinetic does not differ significantly from ZIKA–WT, determined by qPCR. Also, no significant difference in viral titer between ZIKA-EGFP and ZIKA-WT (*p* = 0.113) at 72 h post-infection. Moreover, ZIKA–EGFP was cytopathogenic and led to cytopathic effects, determined with CCK-8 test but weaker than ZIKA–WT. ZIKA-EGFP does not form plaque in Vero cells, but apparent cell clumps were observed under fluorescent microscopy, that consisted with the report, the mechanism is not clear. These findings indicated the effectiveness of constructing a full-length infectious clone of ZIKA–EGFP in a eukaryotic system. Furthermore, we carried out (1) that *EGFP* insertion at positions C36 and C38 also produced stable expression clones; (2) that the vector constructed using this method contained two cyclization sequences (CS) sites. Introduction of a mutation into the second CS site resulted in no change in viral titer; (3) that insertion of Renilla luciferase at position C37 resulted in stable expression for at least three generations (data not shown). Unfortunately, we could not demonstrate the stability of recombinant virus containing EGFP in vivo.

To validate the use of ZIKA–EGFP for monitoring drug effectiveness, we performed an experiment using CAPE, which was shown to inhibit Zika viral replication in our previous experiments. The mechanism of CAPE’s inhibition effect on Zika virus replication which we are working on is not clear. We used CAPE only as a model to prove the ZIKA-EGFP to be effective for drug study. The results showed that treatment with the drug significantly suppressed EGFP expression, which was consistent with results observed following inhibition of RNA replication. These findings suggested that ZIKA–EGFP could be applicable for use in drug screening.

## Conclusions

In summary, we successfully constructed an infectious clone of the full-length genome of Zika virus stably expressing an EGFP reporter in a eukaryotic expression system. This system can be used to study gene function and for antiviral drug screening.

## Supplementary Information

**Additional file 1**: S1 Sequences of 2A peptide, intron, etc.

## Data Availability

Not applicable.

## Abbreviations

NS: Non-structural
E: Envelop
EGFP: Green fluorescence protein
DMEM: Dulbecco’s modified Eagle’s medium
FBS: Fetal bovine serum
PE: Phycoerythrin
ECL: Enhanced chemiluminescence
HRP: Horseradish Peroxidase
PVDF: Polyvinylidene difluoride
BSA: Bovine serum albumin
MOI: Multiplicity of infection
CMV: Cytomegalovirus
HDV: Hepatitis delta virus
qPCR: Fluorescence-based quantitative PCR
TCID50: 50% Tissue culture infectious dose
CCK-8: Cell Counting Kit-8
CAPE: Caffeic Acid Phenethyl Ester
CS: Cyclization sequences
