# Supplementary material for: Construction of an infectious clone of Zika virus stably expressing an EGFP marker in a eukaryotic expression system
Source: Virol J. 2021 Jul 19;18:151. doi: 10.1186/s12985-021-01622-z (PMC8287661; doi:10.1186/s12985-021-01622-z)
Supplement: Supplementary file 1 — Additional file 1: S1 Sequences of 2A peptide, intron, etc. [file 12985_2021_1622_MOESM1_ESM.docx]

Supplemental file1

>2A peptide sequence 54bp

GAGGGCAGAGGAAGTCTTCTAACATGCGGTGACGTGGAGGAGAATCCCGGCCCT

>intron sequence 133bp

GTAAGTATCAAGGTTACAAGACAGGTTTAAGGAGACCAATAGAAACTGGGCTTGTCGAGACAGAGAAGACTCTTGCGTTTCTGATAGGCACCTATTGGTCTTACTGACATCCACTTTGCCTTTCTCTCCACAG

>HDV ribozyme sequence 84bp

GGCCGGCATGGTCCCAGCCTCCTCGCTGGCGCCGGCTGGGCAACATTCCGAGGGGACCGTCCCCTCGGTAATGGCGAATGGGAC

>bGH polyA signal sequence 253bp

CGACTGTGCCTTCTAGTTGCCAGCCATCTGTTGTTTGCCCCTCCCCCGTGCCTTCCTTGACCCTGGAAGGTGCCACTCCCACTGTCCTTTCCTAATAAAATGAGGAAATTGCATCGCATTGTCTGAGTAGGTGTCATTCTATTCTGGGGGGTGGGGTGGGGCAGGACAGCAAGGGGGAGGATTGGGAAGACAATAGCAGGCATGCTGGGGATGCGGTGGGCTCTATGGCTTCTGAGGCGGAAAGAACCAGCTG
